# First report of extended-spectrum beta lactamase (ESBL) and carbapenemase-producing MDR *Klebsiella pneumoniae* from Fuchka

**DOI:** 10.1371/journal.pone.0341583

**Published:** 2026-01-30

**Authors:** Bushra Benta Rahman Prapti, Md. Tanjir Ahmmed, Aminur Rahman, Chandra Shaker Chouhan, Mahbubul Pratik Siddique

**Affiliations:** 1 Department of Microbiology and Hygiene, Faculty of Veterinary Science, Bangladesh Agricultural University, Mymensingh, Bangladesh; 2 Department of Medicine, Faculty of Veterinary Science, Bangladesh Agricultural University, Mymensingh, Bangladesh; Yamagata University Faculty of Medicine: Yamagata Daigaku Igakubu Daigakuin Igakukei Kenkyuka, JAPAN

## Abstract

Extended-spectrum beta-lactamase (ESBL) and Carbapenemase-producing Gram-negative bacteria, particularly *Klebsiella pneumoniae* (ESBL-KP and CP-KP), in the food supply chain, poses a significant public health threat. Ready to eat (RTE) street foods, especially fuchka, a highly popular snack in Bangladesh, India and Southeast Asia, represents a critical breach in food safety. This study investigated the multidrug resistance (MDR) patterns of *K. pneumoniae* isolated from fuchka with an emphasis on phenotypic and genotypic detection of ESBL-KP and CP-KP. A total of 60 samples were collected from 15 fuchka selling points. *K. pneumoniae* was isolated and identified via staining, cultural methods, PCR and MALDI-ToF-MS biotyper. Antibiotic susceptibility test (AST) was accomplished using disk diffusion method, phenotypic ESBL producers was detected by combined disk test (CDT) and PCR was used to detect resistance determinant. Thirty *K. pneumoniae* isolates were confirmed by PCR and MALDI-ToF-MS. All showed resistance to amoxicillin and cefuroxime (100%), while most were sensitive to cefepime (96.7%), norfloxacin (96.7%), imipenem (93.3%) and meropenem (83.3%). All the isolates were MDR, with multiple-antibiotic resistance (MAR) index values ranged from 0.28 to 0.64. CDT confirmed 28 ESBL producers. Among ESBL-producing genes *bla*_TEM,_ was most prevalent (64%), followed by *bla*_SHV,_ *bla*_OXA-1,_ *bla*_CTX-M1,_ *bla*_CTX-M3._ Among carbapenem-resistant genes, *bla*_BIC_ was most common (46%), while *bla*_VIM_ was absent. Moreover, other resistance determinants (*aad*A1, *aac*3IV was most prevalent (40%) but *aad*A1, *qnr*A were not detected. The presence of multidrug-resistant, ESBL- and carbapenemase-producing *K. pneumoniae* in fuchka represents a critical threat to public health.

## 1. Introduction

*Klebsiella pneumoniae* (*K. pneumoniae*), a Gram-negative, atrichous, capsule-forming rod under the Enterobacteriaceae family, is a well-recognized nosocomial bacterial agent responsible for extraintestinal infection in humans and animals [1]. However, *K. pneumoniae* is also associated with foodborne illness, as it is frequently isolated from various food items, such as raw vegetables, ready-to-eat (RTE) foods, even fish, and meat of animal and poultry origin [1,2]. Among Gram-negative bacteria, *K. pneumoniae* has particular importance for its drug-resistant nature from any sources, clinical or food sources [3,4]. More importantly, the foodborne *K. pneumoniae* isolates were found be resistant to several antibiotic classes [5]. The resistance or susceptibility patterns of *K. pneumoniae* comply with recognized categories of drug resistance patterns, viz., multidrug-resistant (MDR; resistance to at least one agent of ≥3 antimicrobial classes), extensively drug-resistant (XDR; except two or fewer classes, resistant to at least one agent in all classes), and pandrug-resistant (PDR; resistant to all of the agents of all classes) [6]. Besides clinical aspects, drug-resistant and MDR *K. pneumoniae* presence in the food chain and its crucial role in aggravating antimicrobial resistance and virulence are serious food safety and public health issues [7].

In fact, the South Asian and other low- and middle-income countries are increasingly impacted by multidrug-resistant organisms, particularly those producing extended-spectrum β-lactamases (ESBLs), a phenomenon largely driven by the irrational use of antibiotics [8]. ESBLs are variants of β-lactamases, enzymes that are capable of efficiently breaking down all penicillins, third- and fourth-generation cephalosporins, and monobactams [9]. These enzymes are typically encoded by genes found in bacteria (most commonly CTX-M, SHV, and TEM; others are OXA, GES, and PER) and are a leading cause of resistance among Enterobacteriaceae species [10]. The complex and charismatic evolutionary history and epidemiological aspects attribute the ESBL-producing isolates as a major public health threat worldwide since the first report in 1983 [9]. More importantly, *K. pneumoniae* is a well-recognized major ESBL-producing bacterium [11].

However, the emergence of multi-drug resistance and ESBL bacterial isolates was resolved when the clinically proven carbapenem, imipenem, was discovered in 1985 [12]. The carbapenem is a broad-spectrum antibiotic, even considered a “last line drug,” against several complex clinical cases caused by MDR and ESBL-producing bacteria [13,14]. Unfortunately, the resistance against carbapenem, ertapenem, had been reported in the early 1990s, and the first report of carbapenem-resistant *K. pneumoniae* was found in Japan, and the first KPC-producing isolates were from the USA [10]. In Europe, particularly in countries like Italy, Greece, and Turkey, carbapenemase-producing Enterobacteriaceae (CPE) have become endemic, with significant numbers of infections originating from local sources [15]. Along with other carbapenem-resistance mechanisms, carbapenemase production, particularly by *K. pneumoniae*, confers resistance to almost all beta-lactam antibiotics [16], which significantly limited therapeutic options to treat multidrug-resistant (MDR) *K. pneumoniae* infections [17].

The carbapenemase enzymes were classified, according to the Ambler molecular classification system, into Class A, Class B, and Class D [18]. The Ambler Class A carbapenemase (also called classical carbapenemase; mediates serine-directed hydrolysis) includes (1) chromosomally encoded enzymes, viz., NmcA (non-metalloenzyme carbapenemase A), SME-1 (Serratia marcescens enzyme-1), SFC-1 (Serratia fonticola carbapenemase-1), SHV-38 (sulfhydryl variable-38), and PenA (penicillin-binding protein A); and (2) plasmid-encoded enzymes, viz., KPC (*Klebsiella pneumoniae* carbapenemase), IMI (imipenem-hydrolyzing beta-lactamase), and varieties of GES (Guiana extended spectrum) [19]. The Ambler class B (metallo beta-lactamases; MBL) also includes both chromosomally and plasmid-encoded enzymes; however, the frequently reported are plasmid-encoded, such as VIM (Verona integron-encoded MBL types), IMP (plasmid-mediated imipenemase), SPM-1 (Sao Paulo MBL type-1), NDM (New Delhi MBL type), GIM (German imipenemase), SIM-1 (Seoul imipenemase-1), DIM (Dutch imipenemase), TMB-1 (Tripoli MBL-1), and KHM-1 (Kyorin University Hospital MBL-1) [20]. Finally, the Ambler class D (also called oxacillinase; plasmid-encoded only; preferred substrate oxacillin) represents those enzymes with a highly conserved serine-based structure as an active site [21]. The carbapenem-hydrolyzing driven enzymes include groups OXA-23-like, OXA-48-like, OXA-58-like, OXA-153-like, and OXA-235. Among the class members, OXA-48-producing *K. pneumoniae* is frequently reported from various country origin, such as Turkey, North America, Japan, Iran, Taiwan, and many other countries [22–26].

Several researches clearly demonstrated that the presence of *K. pneumoniae* in food sources is a potential threat to public health and can be transmitted to humans through various food ingredients at each step from preparation to consumption [27]. Moreover, drug-resistant or multi-drug-resistant *K. pneumoniae* in food sources, regardless of pathogenicity, can play its role as a resistance gene reservoir for humans, the environment, or animals [7]. Due to social and cultural changes, ready-to-eat (RTE), particularly street foods, became the common food item in many countries and varies according to the country’s geography and resources [28]. There are more than a hundred street food items; however, fuchka (fried crisp, hollow ball filled with boiled chickpeas and smashed potatoes, covered with sliced cucumber and onion salad and shredded eggs, and finally served with pouring spicy tamarind water) is the most preferred and highly consumed item in Bangladesh and the Indian subcontinent [29]. Unfortunately, the street food system is the weakest point while considering the food safety issue [30,31], and the highest prevalence of MDR microorganisms can be found in fuchka samples [32].

Over the past decades, numerous alarming reports have documented the prevalence and distribution of multidrug-resistant (MDR) *K. pneumoniae* in both hospital and community settings. However, limited studies have investigated MDR *K. pneumoniae* in food sources, particularly in ready-to-eat items, like fuchka. In developing countries like Bangladesh, where hygiene practices during food preparation are often insufficient, the risk of MDR bacterial contamination in food raises significant public health concerns. This study aims to analyze the multidrug resistance patterns of *K. pneumoniae* isolated from fuchka, with a focus on the molecular detection of extended-spectrum beta-lactamase (ESBL) and carbapenemase-producing strains, as well as the identification of associated resistance genes.

## 2. Materials and methods

### 2.1. Sample collection

80 samples (n = 20, crispy fuchka ball with toppings; n = 20, salad; n = 20, hand wash of seller; n = 20, dish wash water) of famous street food “fuchka” were collected from the Mymensingh City Corporation area, Bangladesh, during the period of November 2023 to March 2024. The chosen study site was categorized into street vendor, shop based, and restaurant since fuchka are now available at different types of food selling points. A verbal consent was taken from the owner of the fuchka selling point; however, a standard consent form was supplied to them. All the samples were properly labelled and kept in ice box. A cold chain was maintained from sample collection to transportation to the laboratory. The experimental steps and detail a procedure was ethically approved by the Experimentation Ethics Committee of the Bangladesh Agricultural University, Mymensingh [Approval No.: AWEEC/BAU/2023(46), Dated: 13.11.2023].

### 2.2. *Isolation and identification*

About 15 g of mashed solid sample (crispy fuchka ball with toppings, salad) and 5 ml of liquid sample (hand wash of seller, dish wash water) were aseptically transferred into sterilized nutrient broth (Himedia, India) and incubated for 24 hr at 37°C for enrichment. After initial enrichment, MacConkey agar plates (Himedia, India) were streaked with a loopful of enriched culture, followed by overnight incubation at 35–37°C for 18–24 h. The colony morphology was observed, and the desired colony (characteristics pink and mucoid colony, indicating lactose fermentation) of *K. pneumoniae* was chosen [33].

### 2.3. PCR assay and MALDI-ToF-MS of Klebsiella pneumoniae

All culture-positive isolates were subjected to molecular detection methods. For polymerase chain reaction (PCR), bacterial genomic DNA extraction was accomplished using the boiling method [34], followed by quantity determination and quality assessment using NanoDrop^TM^ (Thermo Fisher Scientific, USA). The *gyr*A gene sequence primer [35] was used to detect *Klebsiella* spp. at the genus level, and *rpo*B gene sequence [36] used to detect the species level as *K. pneumoniae*. The oligonucleotide sequence and the thermal conditions for PCR mentioned in **Table 1**.

**Table 1:** List of primer with oligonucleotide sequence, amplicon size with reference (temp: temperature; s: seconds; m: minutes; bp: base pair).

Gene	Primer sequence (5`-3`)	Denaturation		Annealing		Elongation		Product size	Reference
		Temp	Time	Temp	Time	Temp	Time		
***Klebsiella* spp. and *K. pneumoniae* PCR detection**									
*gyr*A	F: CGCGTACTATACATGAACGTA	94°C	45 s	57°C	45 s	72°C	50 s	441 bp	[35]
	R: ACCGTTGATCACTTCGGTCAGG								
*rpo*B	F: CAACGGTGTGGTTACTGACG	94°C	30 s	58°C	30 s	72°C	30 s	108 bp	[36]
	R: TCTACGAAGTGGCCGTTTTC								
**ESBL producing genes**									
*bla*_TEM_	F: ATCAGCAATAAACCAGC	95°C	1 m	56°C	1.5 m	72°C	1 m	516 bp	[37]
	R: CCCCGAAGAACGTTTTC								
*bla*_SHV_	F: AGGATTGCATGCCTTTTTG	95°C	1 m	58°C	1 m	72°C	1 m	392 bp	
	R: ATTTGCTGATTTCGCTCG								
*bla*_OXA-1_	F: ATATCTCTACTGTTGCATCTCC	95°C	1 m	58°C	1 m	72°C	1 m	619 bp	
	R: AAACCCTTCAAACCATCC								
*bla*_CTX-M1_	F: GACGATGTCACTGGCTGAGC	95°C	1 m	58°C	1 m	72°C	1 m	500 bp	
	R: AGCCGCCGACGCTAATACA								
*bla*_CTX-M3_	F: CGCTTTGCCATGTGCAGCACC	95°C	1 m	58°C	1 m	72°C	1 m	300 bp	
	R: GCTCAGTACGATCGAGCC								
**Carbapenemase gene**									
*bla*_IMP_	F: GGAATAGAGTGGCTTAAYTCTC	94°C	30 s	52°C	40 s	72°C	50 s	232 bp	[38]
	R: GGTTTAAYAAAACAACCACC								
*bla*_VIM_	F: GATGGTGTTTGGTCGCATA							390 bp	
	R: CGAATGCGCAGCACCAG								
*bla*_SPM_	F: AAAATCTGGGTACGCAAACG							271 bp	
	R: ACATTATCCGCTGGAACAGG								
*bla*_NDM_	F: GGTTTGGCGATCTGGTTTTC	94°C	30 s	58°C	1 m	72°C	1 m	621 bp	[38]
	R: CGGAATGGCTCATCACGATC								
*bla*_OXA-48_	F: CGTCTAGTTCTGCTGTCTTG	94°C	30 s	56°C	1 m	72°C	1 m	438 bp	
	R: CTTGTCATCCTTGTTAGGCG								
*bla*_KPC_	F: GCGTGGTTAAGGATGAACAC	94°C	30 s	56°C	1 m	72°C	1 m	798 bp	
	R: CATCAAGTTCAACCCAACCG								
*bla*_BIC_	F: TATGCAGCTCCTTTAAGGGC	94°C	30 s	52°C	1 m	72°C	1 m	537 bp	
	R: TCATTGGCGGTGCCGTACAC								
**Other resistance gene**									
*aad*A1	F: TATCAGAGGTAGTTGGCGTCAT	94°C	30 s	58°C	1 m	72°C	1 m	484 bp	[39]
	R: GTTCCATAGCGTTAAGGTTTCATT								
*aac*(3)IV	F: CTTCAGGATGGCAAGTTGGT	94°C	1 m	55°C	1 m	72°C	1 m	286 bp	
	R: TCATCTCGTTCTCCGCTCAT								
*qnr*A	F: GGGTATGGATATTATTGATAAAG	94°C	30 s	52°C	1 m	72°C	1 m	670 bp	
	R: CTAATCCGGCAGCACTATTTA								
*qnr*B	F: GGMATHGAAATTCGCCACTG	95°C	1 m	55°C	1 m	72°C	1 m	264 bp	
	R: TTTGCYGYYCGCCAGTCGAA								
*qnr*S	F: GCAAGTTCATTGAACAGGGT	94°C	30 s	62°C	1 m	72°C	1 m	428 bp	
	R: TCTAAACCGTCGAGTTCGGCG								
*sul*2	F: CGGTCCGGCATCCAGCAATCC	94°C	30 s	64°C	30 s	72°C	40 s	293 bp	
	R: CGAGAGCCACGACCGCGCC								

For MALDI-ToF-MS biotyping, the PCR-positive isolates were further grown onto MacConkey agar medium, and maximum 14–16 hr old culture plates were aseptically wrapped with parafilm and placed in an apparently sterile plastic box and transported to the Quality Control Laboratory for Livestock and Livestock Products, Department of Livestock Services, QC Lab Building, Anwar Jang Road, Savar, Dhaka-1343. Identification scores >2.0 confirmed species-level identification.

### 2.4. Antimicrobial susceptibility testing (AST)

Antibiotic susceptibility was determined following the disc diffusion method [40] on Muller-Hinton agar (MHA) (HiMedia, Mumbai, India). A total of 14 commercially available antibiotic disc (Oxoid, Germany, and Bioanalyse, Mumbai, India) were tested, viz., amoxicillin (AMX, 25 µg), amikacin (AK, 30 µg), amoxicillin-clavulanic acid (AMC, 20/10 µg), ceftazidime (CAZ, 30 µg), ceftriaxone (CRO, 30 µg), cefpodoxime (CPD, 10 µg), cefuroxime (CXM, 30 µg), cefepime (FEP, 30 µg), co-trimoxazole (SXT, 25 µg), norfloxacin (NOR, 10 µg), gentamicin (CN, 10 µg), imipenem (IMP, 10 µg), meropenem (MEM, 10 µg), nalidixic acid (NA, 30 µg). The zone of inhibition was measured in millimetres (mm) and interpreted according to Clinical and Laboratory Standards Institute (CLSI) guidelines [41]. The multidrug resistance (MDR) patterns (resistance against at least one agent of ≥ 3 antibiotic classes) of all isolates were assessed following criteria, described by Magiorakos et al. [42]. Moreover, the multiple antibiotic resistances (MAR) index values were calculated following the description of Krumperman [43], where a MAR index ≥ 0.2 indicates isolates originating from high-risk sources [44].

### 2.5. Phenotypic confirmation of ESBL-producers

*Klebsiella pneumoniae* isolates resistant to third generation cephalosporin- (3GC-R) were further screened for ESBL production through combination disk test (CDT) according to the standard protocol [45]. In the CDT, ceftazidime (30 µg/disc; Oxoid Inc., Canada) alone and in combination with clavulanic acid (ceftazidime/clavulanic acid; CZC 40 μg/disc; 30 µg ceftazidime/10 μg clavulanic acid; Oxoid Inc., Canada) were used as recommended by Giske et al. [46]. The MHA plates were inoculated with the tested isolates suspension (0.5 McFarland standard), followed by incubation at 37°C overnight (18 hrs). Finally, the ESBL- producer was confirmed by comparing the diameter of the zone of inhibition for ceftazidime alone to that for the combined disc, where a diameter >5 mm for the combined antibiotic than for ceftazidime alone indicated the positive isolate [46].

### 2.6. Screening and confirmation of ESBL and carbapenemase producing genes

Both uniplex and multiplex PCR assays were performed to determine the presence of β-lactamase encoding genes in the *K. pneumoniae* isolates. Uniplex PCR was used to detect carbapenemase genes (*bla*_NDM_, *bla*_KPC_, *bla*_OXA-48_, and *bla*_BIC_) and ESBL genes (*bla*_TEM_, *bla*_SHV_, *bla*_OXA-1_, *bla*_CTX-M1_, and *bla*_CTX-M3_). Multiplex PCR was perfomed to screen additional carbapenemase genes (*bla*_IMP_, *bla*_VIM_, and *bla*_SPM_). Primer sequences and thermal cycling conditions were adopted from previously published studies [37,38] and are represented in **Table 1**.

### 2.7. AMR resistance gene detection

Resistance genes *aac*(3) IV, *aad*A1, *qnr*A, *qnr*B, *qnr*S, and *sul*2 were screened using the Gene Amp PCR System using specific primer pairs (**Table 1**). All targets were amplified individually using uniplex PCR assay.

### 2.8. Statistical analysis

All data was statistically analysed using SPSS software version 26.0 and R programming. Pearson correlation and Chi-square test was performed to assess relationships between genotypic and phenotypic data. Undefined values were set as (-) in the output file. A Chi-square test was also conducted to evaluate the association between carbapenemase genes and sample sources, as well as to analyse the distribution of ESBL genes across sources. Cochran’s Q test was done to observe significant differences among the frequencies of resistance genes. Additionally, Pearson correlation tests were applied to determine the correlation between antibiotic resistance and sample sources and between carbapenemase genes and antibiotic resistance. Heatmaps were generated using the heatmap package, to represent the strength and direction of correlations and dendrogram. Any NA or infinite values in the matrix were replaced with zero to maintain the completeness of the heatmap.

## 3. Result

### 3.1. Demographic data

**Fig 1:**
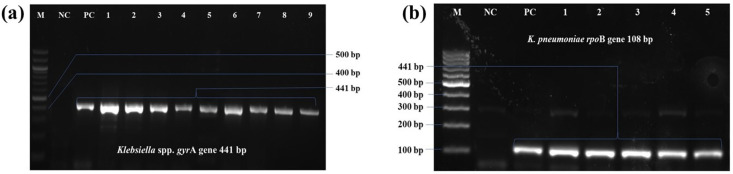
PCR detection of *Klebsiella* spp., targeting *gyr*A gene and *K. pneumoniae*, targeting *rpo*B gene. (a) *Klebsiella* spp. consistent band pattern at 441 bp indicating positive isolates; (b) *K. pneumoniae*, consistent amplicon of 108 bp indicated positive isolates. In both cases, M: 100 bp molecular marker; NC: negative control; PC: positive control.

A total of 30 *Klebsiella pneumoniae* isolates were confirmed by observing cultural and morphological characteristics (S1 Table, S1 Fig, S2 Fig), PCR (**Fig 1**) and MALDI-TOF MS. 13% from (crispy fuchka ball with toppings), 33.3% from (salad), 26.6% from (seller handwashing), and 26.6% (dish washings). All PCR-positive *K. pneumoniae* isolates were confirmed by MALDI-TOF MS with identification scores ranging from 2.01 to 2.23, supporting accurate species-level identification. A chi-square test was conducted to determine whether there was a significant association between the type of outlet (restaurant, shop, or street vendor) and the source of *Klebsiella pneumoniae* contamination. Although variation was observed in contamination sources, shops had the highest number of positive samples (53.3%), followed by vendors (26.7%) and restaurants (20.0%). The association between outlet type and sample source was not statistically significant (χ² = 9.179, p = 0.327).

### 3.2. Phenotypic antibiotic susceptibility test and MDR pattern of K. pneumoniae isolates

**Fig 2:**
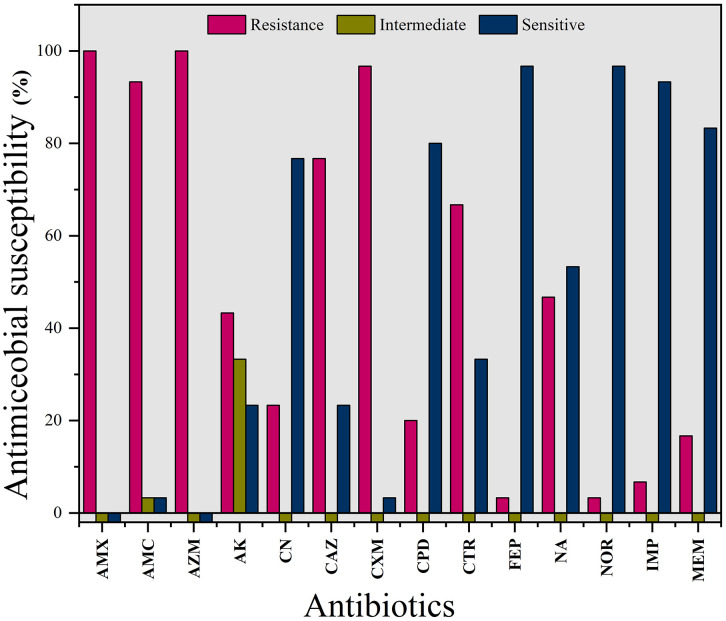
Antibiotic susceptibility percentage of the *K. pneumoniae* isolates from fuchka samples. AMX: Amoxicillin; AMC: Amoxicillin and clavulanic acid; AZM: Azithromycin; AK: Amikacin; CN: Gentamicin; CAZ: Ceftazidime; CXM: Cefuroxime; CPD: Cefpodoxime; CTR: Ceftriaxone; FEP: Cefepime; NA: Nalidixic acid; NOR: Norfloxacin; IMP: Imipenem; MEM: Meropenem.

**Fig 3:**
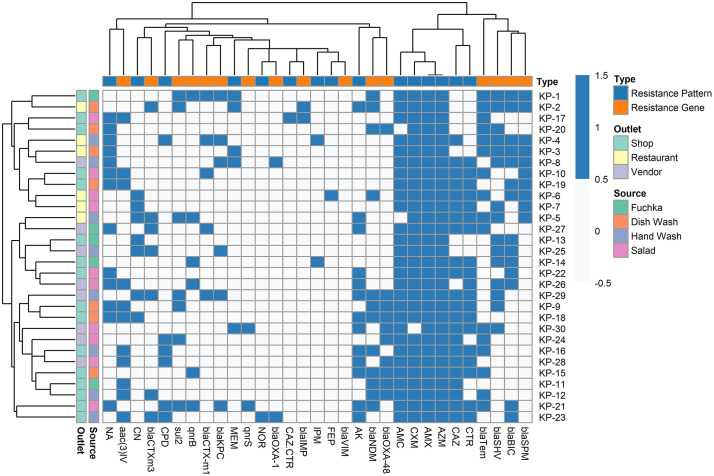
Hierarchical clustering heatmap of antimicrobial resistance genes and phenotypic resistance patterns in *Klebsiella pneumoniae* isolates. AMX: Amoxicillin; AMC: Amoxicillin and clavulanic acid; AZM: Azithromycin; AK: Amikacin; CN: Gentamicin; CAZ: Ceftazidime; CXM: Cefuroxime; CPD: Cefpodoxime; CTR: Ceftriaxone; FEP: Cefepime; NA: Nalidixic acid; NOR: Norfloxacin; IMP: Imipenem; MEM: Meropenem.

*Klebsiella pneumoniae* isolates exhibited complete resistance (100%) to the β-lactam antibiotic amoxicillin, while 93.3% were resistant to amoxicillin-clavulanic acid and 70% to amikacin. Among the cephalosporin class, all isolates (100%) were resistant to cefuroxime, with resistance rates of 76% for ceftazidime and 66.7% for ceftriaxone. However, high sensitivity was observed for cefepime (96.7%) and cefpodoxime (76.7%). In the quinolone class, 96.7% of isolates were sensitive to norfloxacin, although 46.7% demonstrated resistance to nalidixic acid. Within the carbapenem group, sensitivity rates were 93.3% for imipenem and 83.3% for meropenem (Fig 2). Moreover, the similarity relationship among the individual isolates based on their antibiotic susceptibility patterns has been illustrated in **Fig 3** using a hierarchical clustering dendrogram.

The Pearson correlation coefficients between antibiotic resistance phenotypes (R, I, S) across four sample sources: fuchka, dish washings, hand washings, and salad, although most correlations were not statistically significant (*p* > 0.05). Notably, CAZ and CPD showed moderate positive correlation with resistance (r = 0.31, p = 0.10, r = 0.35, p = 0.06) and corresponding negative correlation with susceptibility. (r = −0.31, p = 0.10). In contrast, carbapenem (IPM and MEM) exhibited weak negative correlations with resistance and positive correlation with susceptibility, suggesting minimal association. Other antibiotics showed weak and negligible relationships with sample types (S2 Table).

All fuchka derived isolates of *K. pneumoniae* were found multi-drug resistant (MDR), with 15 isolates (50%) were resistance to four antibiotic classes and 12 (40%) isolates resistant to five antibiotic classes. Penicillin, cephalosporin, and macrolide classes of antibiotics were most commonly resisted (**Table 2**). The multiple antibiotic resistance (MAR) index values ranged from 0.28 to 0.64 (**Table 2**).

**Table 2:** MAR and MDR pattern analysis of the *K. pneumoniae* isolates.

MAR Index	Percentage (%) of isolate	Antibiotic resistance (Class)	No. of isolate	Strand point
.28	3.3%	Penicillin, Cephalosporin, Macrolide	3	MDR
.35	16.67%	Penicillin, Cephalosporin, Macrolide, Carbapenem	2	MDR
.42	6.67%	Penicillin, Cephalosporin, Macrolide, Quinolone	6	MDR
.50	30.0%	Penicillin, Aminoglycoside, Cephalosporin, Macrolide	7	MDR
.57	33.3%	Penicillin, Cephalosporin, Macrolide, Carbapenem, Quinolone	3	MDR
.64	10%	Penicillin, Aminoglycoside, Cephalosporin, Macrolide, Carbapenem	2	MDR
		Penicillin, Aminoglycoside, Cephalosporin, Macrolide, Quinolone	7	MDR

### 3.3. Prevalence of ESBL and carbapenemase producing genes and other ARGs

**Fig 4:**
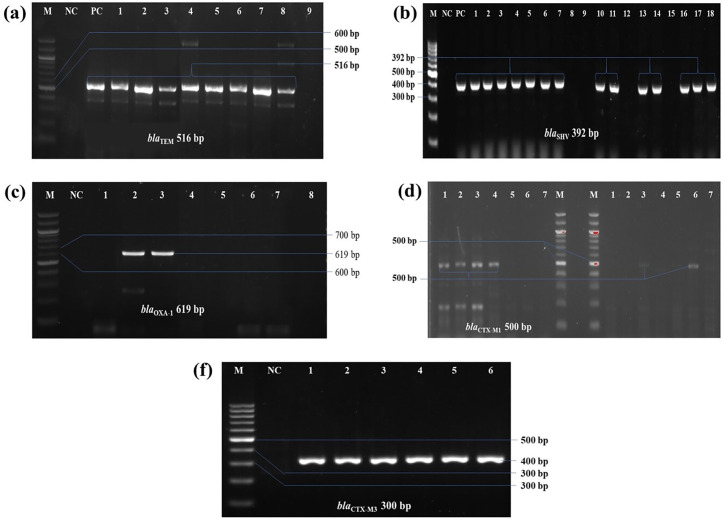
PCR detection of ESBL-producing genes in *K. pneumoniae* isolates. **(a)** *bla*_TEM_ gene, 516 bp; **(b)** *bla*_SHV_ gene, 392 bp; **(c)** *bla*_OXA-1_ gene, 619 bp; **(d)** *bla*_CTX-M1_ gene, 500 bp; and **(e)** *bla*_CTX-M3_, 300 bp. In all cases, M: 100 bp molecular marker; NC: negative control; PC: positive control.

**Fig 5:**
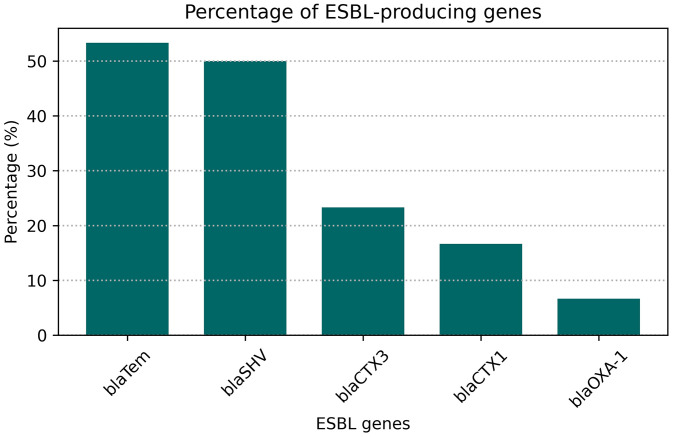
Occurrence (%) of ESBL genes in *K. pneumoniae* isolates from fuchka. Differences in gene frequencies were significant (Cochran’s Q test, p < 0.001).

In the CDST test, n = 28 among 30 (93.3%) *K. pneumoniae* isolates were phenotypically identified as ESBL producers (S3 Fig). Subsequently, all (n = 30) isolates were then subjected to molecular detection of beta-lactamase encoding genes, and 83.3%, n = 25 isolates found positive for at least one tested ESBL producing gene (**Figs 3 and 4**). Highest prevalent ESBL determinant gene in isolated *K. pneumoniae* was *bla*_TEM_ found in 64% (16/25) of the isolates. Other ESBL producing gene patterns with respect to *bla*_CTX-M1_, *bla*_CTX-M3_, *bla*_SHV_, and *bla*_OXA-1_ is given in (Fig 5). Cochran’s Q test revealed a statistically significant difference in the detection frequencies among the ESBL genes (Q(4) = 21.18, p < 0.001), indicating that not all ESBL gene types were equally prevalent among the isolates.

Co-existence of multiple ESBL-producing genes was observed in 23.3% (7/30) of the isolates, which harboured both the *bla*_TEM_ and *bla*_SHV_ genes. The various co-occurrence patterns of these ESBL genes are detailed in (Table 3**).** The ESBL genes revealed significant associations for *bla*_CTX-M3_ with dish wash samples (χ² = 4.5, p = 0.03) and *bla*_CTX-M1_ with salad samples (χ² = 6.4, p = 0.01). *bla*_OXA-1_ displayed a marginal association with hand wash samples (χ² = 2, p = 0.16), whereas the *bla*_TEM_ and *bla*_SHV_ genes showed no significant relationships with any sample source (p > 0.05) (Table 4).

**Table 3:** Co-existence of ESBL and carbapenemase producing multiple resistant genes in *K. pneumoniae* isolates detected from fuchka samples.

ESBL gene positive isolate 83.3% (n = 25)	Co-existence of ESBL positive genes	Prevalence (%)
*bla*_TEM_ and *bla*_SHV_	7 (28)
*bla*_TEM_ and *bla*_CTX-M1_	3 (12.0))
*bla*_TEM_ and *bla*_CTX-M3_	4 (16.0)
*bla*_SHV_ and *bla*_OXA-1_	1 (4.0)
*bla*_SHV_ and *bla*_CTX-M1_	3 (12.0)
*bla*_SHV_ and *bla*_CTX-M3_	4 (16.0)
*bla*_OXA-1_ and *bla*_CTX-M3_	1 (4.0)
*bla*_CTX-M1_ and *bla*_CTX-M3_	2 (8.0)
*bla*_TEM_, *bla*_SHV_ and *bla*_CTX-M1_	2 (8.0)
*bla*_TEM_, *bla*_SHV_ and *bla*_CTX-M3_	2 (8.0)
Carbapenemase gene positive isolate 100% (n = 30)	*bla*_NDM_ and *bla*_KPC_	2 (6.7)
	*bla*_NDM_ and *bla*_OXA-48_	7 (23.3)
	*bla*_NDM_ and *bla*_BIC_	4 (13.3)
	*bla*_NDM_ and *bla*_SPM_	3 (10)
	*bla*_NDM_ and *bla*_IMP_	2 (6.7)
	*bla*_KPC_ and *bla*_OXA-48_	1 (3.3)
	*bla*_KPC_ and *bla*_BIC_	3 (10)
	*bla*_BIC_ and *bla*_SPM_	7 (23.3)
	*bla*_NDM_, *bla*_KPC_ and *bla*_OXA-48_	1 (3.3)
	*bla*_NDM_, *bla*_KPC_ and *bla*_BIC_	2 (6.7)
	*bla*_NDM_, *bla*_KPC_ and *bla*_SPM_	1 (3.3)
	*bla*_NDM_, *bla*_BIC_ and *bla*_SPM_	2 (6.7)
	*bla*_KPC_, *bla*_BIC_ and *bla*_SPM_	3 (10)
	*bla*_NDM_, *bla*_KPC_, *bla*_BIC_ and *bla*_SPM_	1 (3.3)

**Table 4:** Association of ESBL- and carbapenemase-encoding genes with different sample sources based on the chi-square (χ²) test.

	Gene	Fuchka	Dish Wash	Hand Wash	Salad
		χ² (p value)	χ² (p value)	χ² (p value)	χ² (p value)
ESBL genes	*bla*_TEM_	0 (1.0)	0 (1.0)	0 (1.0)	0.4 (0.53)
	*bla*_SHV_	0 (1.0)	0 (1.0)	0.5 (0.48)	1.6 (0.21)
	*bla*_OXA-1_	–	–	2 (0.16)	–
	*bla*_CTX-M3_	1 (0.32)	4.5 (0.03)	0.5 (0.48)	–
	*bla*_CTX-M1_	0 (1.0)	–	2 (0.16)	6.4 (0.01)
Carbapenemase genes	*bla*_NDM_	0 (1.0)	0.5 (0.48)	0.5 (0.48)	3.6 (0.06)
	*bla*_KPC_	1 (0.32)	–	0 (1.0)	6.4 (0.01)
	*bla*_IMP_	–	4.5 (0.03)	–	6.4 (0.01)
	*bla*_OXA-48_	1 (0.32)	0.5 (0.48)	2 (0.16)	1.6 (0.21)
	*bla*_BIC_	0 (1.0)	0.5 (0.48)	0 (1.0)	0.4 (0.53)
	*bla*_SPM_	0 (1.0)	0.5 (0.48)	0.5 (0.48)	0.4 (0.53)

**Fig 6:**
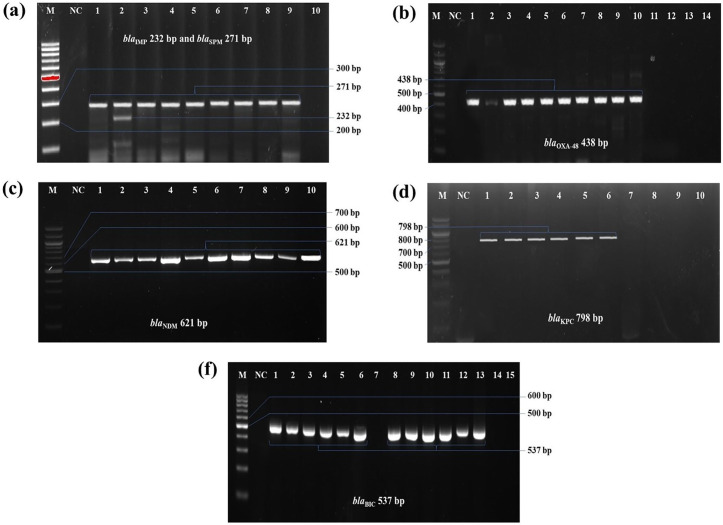
PCR-based detection of carbapenemase producing genes in *K. pneumoniae* isolates. **(a)** *bla*_IMP_ gene, 232 bp and *bla*_SPM_ gene, 271 bp; **(b)** *bla*_OXA-48_ gene, 438 bp; **(c)** *bla*_NDM_ gene, 621 bp; **(d)** *bla*_KPC_ gene, 798 bp; and **(e)** *bla*_BIC_ gene, 537 bp. In all cases, M: 100 bp molecular marker; NC: negative control.

**Fig 7:**
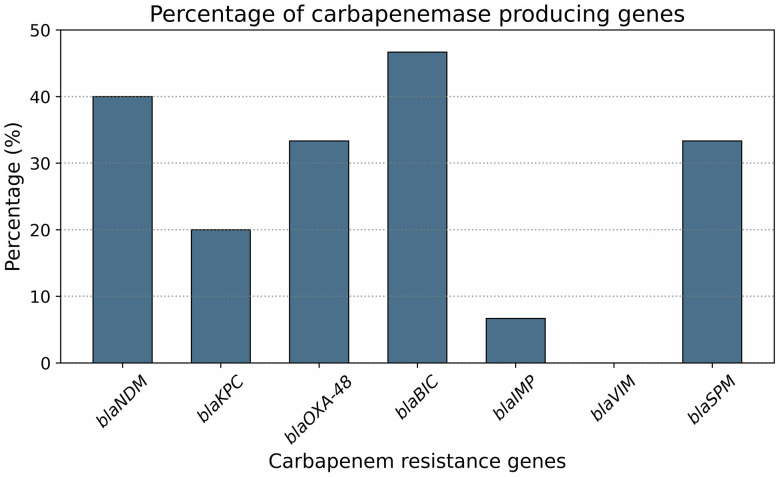
Occurrence percentage of carbapenemase producing gene. Differences in gene frequencies were significant (Cochran’s Q test, p < 0.001).

At least one carbapenemase encoding gene was detected among all *K. pneumoniae* isolates (n = 30). The PCR-based detection of carbapenemase encoding genes is represented in **Fig 6**. The highest prevalence of carbapenemase producers was observed 46% (n = 14) and 40% (n = 12) for *bla*_BIC_ and *bla*_NDM_, respectively, whereas none of the isolates were positive for *bla*_VIM._ Cochran’s Q test revealed a statistically significant difference in the occurrence frequencies among the seven carbapenemase genes (Q(6) = 27.24, p < 0.001), indicating that not all genes were equally prevalent. The distribution of carbapenemase genes in various sample sources is shown in **Fig 3**. Remarkably, only a single isolate (3.3%) harboured four resistance genes (*bla*_NDM_, *bla*_BIC_, *bla*_IMP_, and *bla*_SPM_) together (**Fig 7**). The *bla*_IMP_ gene was significantly associated with dish washings (χ² = 4.5, p = 0.03) and salad (χ² = 6.4, p = 0.01), while the *bla*_KPC_ gene also showed a strong association with salad (χ² = 6.4, p = 0.01). The *bla*_NDM_ gene exhibited a near-significant association with salad samples (χ² = 3.6, p = 0.06). However, no significant associations were observed for the *bla*_OXA-48_, *bla*_BIC_, and *bla*_SPM_ genes across the sample sources (p > 0.05) (Table 4).

**Fig 8:**
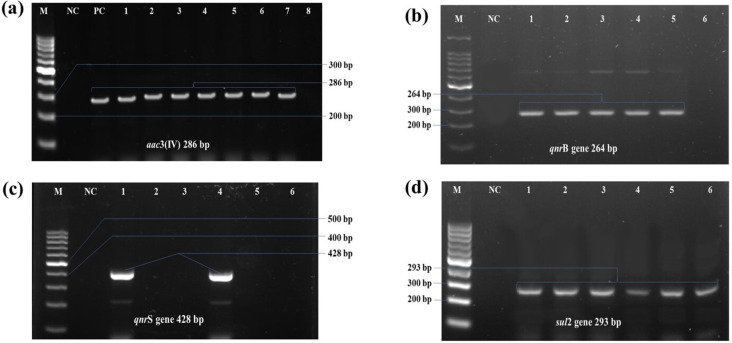
PCR detection of antimicrobial resistance genes (other than ESBL and Carbapenem resistance genes) in *K. pneumoniae* isolates, where the consistent and clear bands at desired position indicated positive results. (a) *aac*3(IV) gene, amplicon size 286 bp; (b) *qnr*B gene, amplicon size 264 bp; (c) *qnr*S gene, amplicon size 428 bp; (d) *sul*2 gene, amplicon size 293 bp. In all cases, M: 100 bp molecular marker; NC: negative control.

Among the aminoglycoside resistance-associated genes, the *aac*3IV was detected in 40% (12/30) of isolates. Sulfonamide resistance gene *sul*2 was found in 23.3% (7/30), and quinolone resistance genes *qnr*B, *qnr*S were found in 16.7% (5/30), and 6.7% (2/30) of isolates, respectively, as reflected in the dendrogram. No *aad*A1or *qnr*A genes were detected in this study. However, among the tested isolates, only one isolate carried both *aac3*IV and *sul*2 genes*,* and another isolate carried both *aac*3IV and *qnr*B genes through PCR. The PCR detection of ARGs has been depicted in **Fig 8**.

### 3.5. Co-relation between phenotypic and genotypic resistance expression

**Fig 9:**
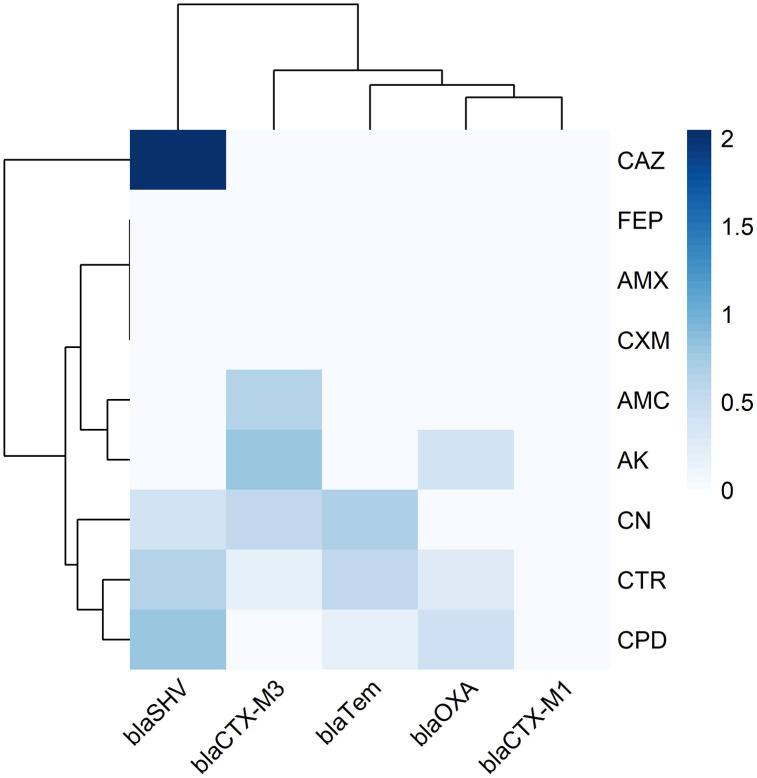
Heatmap of chi-square analysis between ESBL-producing genes and antibiotic resistance phenotypes. **Note: “Blue” color express significant relationship (p < 0.05)**.

The chi-square analysis (**Fig 9**) demonstrated variable association between ESBL genes and antibiotic resistance profiles. A significant association was observed (p < 0.05) between the presence of *bla*_SHV_ gene and susceptibility pattern of ceftazidime (CAZ) resistance, suggesting that *bla*_SHV_ may influence the expression of resistance to this β-lactam antibiotic.. In contrast, no statistically significant association were detected for *bla*_TEM_, *bla*_OXA_, *bla*_CTX-M1_, or *bla*_CTX-M3_ with the other tested antibiotics (*p* > 0.05), indicating a limited phenotypic effect of these genes on resistance under the study conditions.

The heatmap analysis demonstrated clear co-resistance, most frequently observed among antibiotics targeting β-lactamase (*bla*_TEM_, *bla*_SHV_, *bla*_CTX-M_), aminoglycoside-modifying enzyme genes (*aac*(3)-II, *aad*A1), tetracycline resistance genes (*tet*A, *tet*B), and macrolide resistance genes (*erm*B, *mph*A), indicating genetic linkage and possible horizontal gene transfer. Isolates from salad and handwash samples showed a higher prevalence of multidrug resistance, particularly against β-lactams, tetracyclines, and fluoroquinolones, reflecting potential co-selection pressure and environmental dissemination of resistant determinants (Fig 3).

As well as, chi-square analysis between carbapenemase genes and carbapenem resistance antibiotics revealed that most of the association was statistically non- significant (p > 0.05). However, the significant positive association was observed between the *bla*_SPM_ gene and meropenem (MEM) resistance (**χ²** = 5.88, p = 0.015). Other carbapenemase genes exhibited weak or no significant association with carbapenem resistance phenotypes (**Table 5**).

**Table 5:** Association between carbapenemase encoding genes with antibiotic resistance phenotypes.

Carbapenemase genes	IPM		MEM	
	(χ²)	p-value	(χ²)	p-value
*bla*_NDM_	1.43	0.23	0.0	1
*bla*_KPC_	1.21	0.27	1.50	0.22
*bla*_OXA-48_	1.07	0.30	0.48	0.48
*bla*_BIC_	2.45	0.12	2.68	0.11
*bla*_IMP_	0.15	0.69	1.71	0.19
*bla*_SPM_	0.27	0.60	5.88	0.015*

“*” indicates statistically significant association (p < 0.05).

## 4. Discussion

As a foodborne pathogen, *Klebsiella pneumoniae* got the focus in the late 1990s [47]. However, *K. pneumoniae* from food sources, with its virulence and, antimicrobial resistance, particularly ESBL and carbapenemase production capability, came into the spotlight in the late 2010s [7,48–51]. The comparative genomics and comparative antimicrobial resistance patterns of *K. pneumoniae* from clinical and food sources were also reported [52–54] and an overlapping population was found [53]. Among various food sources, *K. pneumoniae* contamination had been reported from ready-to-eat food, particularly street foods, in several countries, such as Malaysia, Indonesia, Nigeria, Ethiopia, Egypt, and even Europe [49,55–59].

Despite this, there is limited [60] or no detail and systematic approach to reveal the antimicrobial resistance and public health impacts of *K. pneumoniae* from food sources in the Indian subcontinent countries, even in South Asian countries. This present study address this gap by characterizing extended-spectrum beta-lactamase (ESBL) and carbapenemase-producing genes in *K. pneumoniae* isolated from fuchka, a popular ready-to-eat (RTE) food. As a pathogenically significant member of the *Enterobacteriaceae* family, *K. pneumoniae* has become increasingly concerning due to its role in disseminating AMR genes over the last few decades. This underscores the urgent need to identify resistance patterns and genes responsible for AMR, such as ESBL and carbapenemase, in RTE foods, since in today’s world, lifestyle and dietary habits heavily rely on various types of RTE foods [61,62].

The presence of *K. pneumoniae* in fuchka, indicates contamination from environmental and human sources and reflects hygiene and sanitary practices from preparation, handling, storage, distribution, and serving to the consumers, as the bacterium is a part of normal human and animal gut microbiota [63]. Although fuchka is traditionally considered as street food item, but in Bangladesh and other Indian subcontinents, it is commonly available in a wide range of food outlets, including dedicated fuchka shops and restaurants. In the present study, the primary isolation and identification of *K. pneumoniae* from fuchka and fuchka-related materials was accomplished using cultural methods, Gram’s staining, PCR and MALDI-ToF-MS. While the cultural characterization was performed, the pink mucoid colonies were observed on EMB agar medium and MacConkey agar medium, which is the characteristic colony morphology of *K. pneumoniae*, as described in another studies [64–66]. While using chromogenic agar medium (HiCrome UTI agar; HiMedia, India), the colonies appeared blue to purple with mucoid colonies, similar to the findings of Khutade et al. [67]. The Gram’s staining revealed Gram-negative rods [68]. The PCR assays consistently produced amplicon at 441 bp [36]. In the case of MALDI-ToF-MS analysis, all the isolates of *K. pneumoniae* were identified clearly (score values ranged from 2.21 to 2.39; secured genus identification) as per the description of Normand et al. [69].

Based on the conventional (cultural and morphological) and molecular detection methods (PCR and MALDI-ToF-MS), a notable prevalence of *K. pneumoniae* was observed in fuchka, particularly from salad samples (33.3%), as well as from sellers’ hand wash and dish washing water (26.6%). Bedair et al. [58] also recovered 31.8% *K. pneumoniae* isolates from 242 green salad samples in Egypt. Giri et al. [60] isolated overall 27.12% *K. pneumoniae* from street foods in India; however, there was a 28.12% isolation rate from pani puri samples. Khalif et al. [32] observed the highest prevalence of *K. pneumoniae* in fuchka, though that was only 2%. Ezemba et al. [57] reported a 20.24% prevalence of *K. pneumoniae* from ready-to-eat foods in Nigeria. In fact, variable occurrence rates of *K. pneumoniae* were also reported from other food samples, such as Guo et al. [5] who found only 9.9% *K. pneumoniae* from raw seafood, raw chicken, frozen raw food and cooked food samples. Some previous studies were conducted on *K. pneumoniae* from raw food and frozen food samples and showed the prevalence of 16.1%, 5.6%, and 9.9% MDR *K. pneumoniae* isolates [70–72].

Regarding the antimicrobial resistance potential of *K. pneumoniae*, Petrosillo et al. stated that 73.1% of *K. pneumoniae* are resistant to at least one antibiotic [73]. In this study, isolates exhibit 100% resistance towards amoxicillin and 93.7% resistance to amoxicillin and clavulanic acid and the least resistance towards meropenem and imipenem, with 100% sensitivity towards norfloxacin and sulfamethoxazole-trimethoprim. Similar findings were observed in other studies conducted on street food [60]. *K. pneumoniae* from RTE food showed higher sensitivity towards, Ciprofloxacin (8%) and sulfamethoxazole-trimethoprim (9%), with 100% sensitivity towards gentamicin and norfloxacin [7]. All *K. pneumoniae* isolates from fuchka samples in this study were (multidrug resistant) MDR and MAR index were valued >0.2. Giri et al. [60] reported a MAR value of 86.44% of isolates as >0.2, and most of the isolates were MDR, while characterizing ESBL producing *E. coli* and *K. pneumoniae* from RTE foods in India. Guo et al. and Bedair et al. reported 19.19% and 27.3% MDR *K. pneumoniae* from RTE foods from China and Egypt, respectively [5,58]. As all isolates in the present study were MDR irrespective of outlet type (street vendor, shop, or restaurant), no statistical correlation could be established between source and AMR contamination. This uniform MDR pattern across outlets suggests that resistance dissemination may be widespread throughout the fuchka production and distribution chain, likely reflecting common contamination routes such as unsafe water, improper handling, or poor hygiene. However, studies from Bangladesh specifically addressing the prevalence of antimicrobial resistance (AMR) and extended-spectrum beta-lactamase (ESBL)-producing *K. pneumoniae* in ready-to-eat (RTE) foods remain scarce, with limited data available globally [74]. The extensive focus on *Escherichia coli* and other major foodborne pathogens across various sources may have contributed to underestimating the role of *K. pneumoniae* as a significant organism in RTE street foods and ESBL and carbapenemase production [58].

In this study, the prevalence of ESBL-producing *K. pneumoniae* was 83.3%. A slight discrepancy was observed between the phenotypic (CDST) and genotypic detection of ESBLs, where 28 isolates were CDST-positive but only 25 carried at least one of the tested ESBL genes. Such differences have been reported previously and may arise due to the production of other β-lactamases (e.g., AmpC or inhibitor-resistant variants) that mimic the ESBL phenotype, or due to the presence of ESBL gene variants not targeted by the selected primers. Additionally, plasmid loss or sequence variation at primer binding sites could contribute to PCR-negative results. These findings highlight the importance of combining phenotypic and molecular methods for accurate ESBL detection. Although limited published data on ESBL-producing *K. pneumoniae* from food sources exists, this finding is consistent with previous reports on hospital-acquired *K. pneumoniae* [75]. Among the total isolates, 64%, 60%, 8%, 20%, and 28% harbored the *bla*_TEM,_ *bla*_SHV_, *bla-*_OXA1_, *bla*_CTX-M1_, and *bla*_CTX-M3_ genes, respectively. In comparison, 46.7% of ESBL-producing isolates in prior studies were found to carry *bla*_TEM_ (34.3%), *bla*_SHV_ (20%), and *bla*_CTX-M_ (5.7%) genes [76]. Both studies show a higher prevalence of *bla*_TEM_ and *bla*_SHV_. While several reports have indicated that *bla*_SHV-12_ and *bla*_CTX-M1_ predominated in food isolates [59], our study found a higher prevalence of *bla*_TEM_ and *bla*_SHV_, similar to a study on ready-to-eat food, which reported the highest prevalence of *bla*_TEM_ (51%) [77]. Notably, *bla*_TEM_ and *bla*_CTX-M_ genes, which are associated with mobile genetic elements such as transposons and plasmids, have been shown to facilitate horizontal gene transfer within and between populations of Gram-negative bacteria [78]. Coexistence of *bla*_TEM_ and *bla*_CTX-M1_, *bla*_CTX-M3_ was observed in 6 ESBL-positive strains. In this study, plasmid-mediated quinolone resistance determinants (*qnr* genes) conferred quinolone resistance in seven (7) *K. pneumoniae* strains. Moreover, *qnr*-positive *K. pneumoniae* isolates were found to harbor at least one ESBL subtype (CTX-M*,* OXA-1*,* TEM*, and* SHV) or *qnr* genes. In fact, the majorities of ESBL- associated genes are transposon- and plasmid-mediated, and confer plasmid-mediated quinolone resistance determinants [75,79].

In the current study, all isolates (100%) found carrying at least one carbapenem-resistant gene was phenotypically susceptible to imipenem and meropenem. A notable finding of this study is that several isolates carrying carbapenemase genes remained phenotypically susceptible to imipenem and meropenem. Similar “silent” or low-expression carriers have been documented, most notably for IMP-6–type enzymes that confer resistance to meropenem but not always to imipenem [80]. Such discordance may result from a variety of mechanisms, including low transcriptional activity of the carbapenemase gene, mutations in regulatory or promoter regions, low plasmid copy number, or the presence of non-functional or weakly active variants. Additionally, intact outer-membrane porins (OmpK35/OmpK36) and the absence of efflux-pump up-regulation can maintain susceptibility even when carbapenemase genes are present.

The widespread occurrence of carbapenemase-encoding genes in foodborne *K. pneumoniae* isolates observed in this study is therefore alarming, as it indicates the potential for horizontal gene transfer of these clinically significant resistance determinants. Similar findings were also reported by Albasha et al. [81].. This confirms that this gene is not stable and relies upon other synergistic mechanisms to mediate resistance against carbapenems [82]. Although six strains of *K. pneumoniae* in this study were moderately sensitive to imipenem and meropenem, 40%, 20%, and 33.3% tested positive for *bla*_NDM_, *bla*_KPC_, *bla*_OXA-48_, which is somewhat different from others [81]. In another study, the occurrence of carbapenemase-producing *K. pneumoniae* from chicken meat in Algeria was 16%, where OXA-48 and NDM-1 producers were 79% and 21%, respectively [72], All isolates detect having a carbapenem resistance gene, where 23.3% (*bla*_NDM_, *bla*_OXA-48_ and *bla*_BIC_, *bla*_SPM_) had multiple genes co-occurring. This finding agrees with others [81,83], which showed a multiplicity of genes in their isolates. Moreover, the metallo-β-lactamase (MBL) gene *bla*_IMP_ was detected in two strains, while ten strains were positive for *bla*_SPM_. A prior investigation on chicken and environmental samples reported 5% positivity for *IMP* and *VIM* genes [84]. However, this study did not identify any *VIM*-positive genes.

A significant association was observed between the ESBL producing gene *bla*_CTX-M1_ and dishwash samples (p-value 0.03), as well as, with salad sample (p-value 0.01). This could be due to improper sanitation practices, or the presence of biofilms on dishwashing equipment or the reuse of contaminated cleaning tools, all of which can facilitate the persistence and spread of antimicrobial resistance genes. This study also found that the carbapenemase genes IMP and both salad and dishwash samples, and between KPC and salad samples. Similarly, the ESBL genes CTXM3 (dishwashing) and CTXM1 (salad) were both significantly associated. These observations align with prior reports [60,85]. This might be due to the potential contamination of salad and dish wash during food preparation or cleaning processes, as well as the fact that salad is often prepared long in advance. The presence of water in salad, which can facilitate the survival and spread of bacteria, might also contribute to the persistence of antimicrobial resistance genes [86,87].

In our isolates, in addition to β-lactamase and carbapenemase genes, determinants for aminoglycoside-modifying enzyme (*acc3*(IV)), sulfonamide-modifying enzyme (*sul*2), and fluoroquinolone efflux pump (*qnr*B) and (*qnr*S) were identified in the multidrug-resistant (MDR) isolates, thereby posing a significant threat to fuchka and other ready-to-eat food consumers. Prevalence of *aac*(3(IV)) 40%, *sul*2 23%, *qnr*B 16.7%, and *qnr*S 6.7% was observed among the isolated strains. 10% *aac3*(IV)*,* 38% *sul*2, 10% *qnr*A and 12% *qnr*B were reported in *K. pneumoniae* from clinical isolates [88]. This report has similarity with our findings except for the prevalence of *qnr*A*,* as no strain in our study was found positive for *qnr*A*.*

There are several limitations in this study that need to be acknowledged. Firstly, the sample size analyzed is relatively small, making it challenging to draw robust conclusions regarding the presence of ESBL and carbapenemase producing *K. pneumoniae* in fuchka (a popular street food). To validate and strengthen these findings, larger-scale studies with a greater number of samples are necessary. Secondly, focusing on just one type of ready-to-eat food does not provide a comprehensive understanding of the prevalence and transmission dynamics of *Klebsiella pneumoniae* and its associated antimicrobial resistance (AMR) genes. A wider variety of food items should be included to capture the full scope of the issue. Additionally, this study did not address potential factors such as food handling practices, storage conditions, or cross-contamination, which could influence the spread of resistant strains. Future research should aim to incorporate a more diverse range of food samples and consider these external factors in order to better understand the role of food in the transmission of AMR.

## 5. Conclusions

To best of our knowledge, this is the first study to report ESBL- and carbapenemase-producing and MDR *K. pneumoniae* from RTE foods, fuchka, from Mymensingh city corporation area, Bangladesh. In conclusion, *bla*_TEM_-type ESBL-producing genes and *bla*_BIC_ and *bla*_NDM_ type carbapenemase are prevalent among the isolates. High resistance found towards cephalosporins, and aminoglycoside. Resistance towards cephalosporins and carbapenems has increased many folds during study period. ESBL- and carbapenemase-producing and MDR *K. pneumoniae* through food chain could be a major public health concern. Regarding microbial food safety in fuchka, it could be concluded hygiene and sanitary practices should strictly be maintained from the preparation stage to the consumer level and regular monitoring and surveillance from the law and enforcement agencies on hygiene and sanitary practices and food safety issues related to fuchka and other food preparation and selling services should be conducted.

## Supporting information

S1 Table Cultural, morphological and biochemical characteristics of *Klebsiella pneumoniae.* (DOCX)

S2 Table Association of antibiotic resistance phenotypes with fuchka, dish wash, hand wash, and salad samples (Pearson’s coefficient test). Pearson’s Coefficient values (p-value). R = resistance, I = intermediate sensitivity, S = sensitive. AMC = amoxicillin, AK = amikacin, CAZ = ceftazidime, CPD = cefpodoxime, NOR= norfloxacin, CTR = ceftriaxone, CXM = cefixime, FEP = cefepime, CN = gentamicin, IPM = imipenem, MEM = meropenem, NA = nalidixic acid.
(DOCX)

S1 Fig Colonies of *K. pneumoniae* on MacConkey agar. (JPG)

S2 Fig Morphological characteristics of *K. pneumoniae* isolates (Gram’s staining). (JPG)

S3 Fig Combined disk test (CDST) to detect ESBL-producer *K. pneumoniae.* (JPG)
