# Supplementary material for: First report of extended-spectrum beta lactamase (ESBL) and carbapenemase-producing MDR Klebsiella pneumoniae from Fuchka
Source: PLoS One. 2026 Jan 30;21(1):e0341583. doi: 10.1371/journal.pone.0341583 (PMC12858000; doi:10.1371/journal.pone.0341583)
Supplement: S1 Table — (DOCX) [file pone.0341583.s001.docx]

**S1 Table**. Cultural, morphological and biochemical characteristics of Klebsiella pneumoniae

| **Parameter** | **Characteristic** |
| --- | --- |
| **Colony Characteristics** | |
| Colony appearance | Large, smooth, distinctly mucoid colonies |
| Colony color | pink on MacConkey agar |
| **Microscopic Features** | |
| Gram staining | Gram-negative |
| Cell shape | Short rod-shaped bacilli |
| **Biochemical Characteristics** | |
| Catalase test | Positive |
| Indole test | Negative |
| MR (Methyl Red) test | Negative |
| VP (Voges-Proskauer) test | Positive |

|  |  |
| --- | --- |
|  |  |
|  |  |
|  |  |
