# Supplementary material for: First report of extended-spectrum beta lactamase (ESBL) and carbapenemase-producing MDR Klebsiella pneumoniae from Fuchka
Source: PLoS One. 2026 Jan 30;21(1):e0341583. doi: 10.1371/journal.pone.0341583 (PMC12858000; doi:10.1371/journal.pone.0341583)
Supplement: S2 Table — R = resistance, I = intermediate sensitivity, S = sensitive. AMC = amoxicillin, AK = amikacin, CAZ = ceftazidime, CPD = cefpodoxime, NOR= norfloxacin, CTR = ceftriaxone, CXM = cefixime, FEP = cefepime, CN = gentamicin, IPM = imipenem, MEM = meropenem, NA = nalidixic acid. (DOCX) [file pone.0341583.s002.docx]

**S2 Table. Association of Antibiotic Resistance Phenotypes with Fuchka, Dish Wash, Hand Wash, and Salad Samples (Pearson's Coefficient test)**

| **Antibiotic** | **Resistance pattern** | **Sample** | | | |
| --- | --- | --- | --- | --- | --- |
|  |  | **Fuchka** | **Dish washings** | **Hand washings** | **Salad** |
| AMC | R | 0.15 (0.43) | 0.15 (0.43) | 0.15 (0.43) | 0.15 (0.43) |
| AMC | I | -0.26 (0.17) | -0.26 (0.17) | -0.26 (0.17) | -0.26 (0.17) |
| AMC | S | 0.05 (0.79) | 0.05 (0.79) | 0.05 (0.79) | 0.05 (0.79) |
| AK | R | 0.13 (0.49) | 0.13 (0.49) | 0.13 (0.49) | 0.13 (0.49) |
| AK | I | -0.04 (0.84) | -0.04 (0.84) | -0.04 (0.84) | -0.04 (0.84) |
| AK | S | -0.11 (0.56) | -0.11 (0.56) | -0.11 (0.56) | -0.11 (0.56) |
| CAZ | R | 0.31 (0.10) | 0.31 (0.10) | 0.31 (0.10) | 0.31 (0.10) |
| CAZ | S | -0.31 (0.10) | -0.31 (0.10) | -0.31 (0.10) | -0.31 (0.10) |
| CPD | R | 0.35 (0.06) | 0.35 (0.06) | 0.35 (0.06) | 0.35 (0.06) |
| CPD | S | -0.35 (0.06) | -0.35 (0.06) | -0.35 (0.06) | -0.35 (0.06) |
| NOR | R | 0.05 (0.79) | 0.05 (0.79) | 0.05 (0.79) | 0.05 (0.79) |
| NOR | S | -0.05 (0.79) | -0.05 (0.79) | -0.05 (0.79) | -0.05 (0.79) |
| CTR | R | 0.28 (0.14) | 0.28 (0.14) | 0.28 (0.14) | 0.28 (0.14) |
| CTR | S | -0.28 (0.14) | -0.28 (0.14) | -0.28 (0.14) | -0.28 (0.14) |
| CXM | R | -0.21 (0.27) | -0.21 (0.27) | -0.21 (0.27) | -0.21 (0.27) |
| CXM | I | 0.21 (0.27) | 0.21 (0.27) | 0.21 (0.27) | 0.21 (0.27) |
| FEP | R | 0.21 (0.27) | 0.21 (0.27) | 0.21 (0.27) | 0.21 (0.27) |
| FEP | S | -0.21 (0.27) | -0.21 (0.27) | -0.21 (0.27) | -0.21 (0.27) |
| CN | R | 0.09 (0.64) | 0.09 (0.64) | 0.09 (0.64) | 0.09 (0.64) |
| CN | S | -0.09 (0.64) | -0.09 (0.64) | -0.09 (0.64) | -0.09 (0.64) |
| IPM | R | -0.15 (0.43) | -0.15 (0.43) | -0.15 (0.43) | -0.15 (0.43) |
| IPM | S | 0.15 (0.43) | 0.15 (0.43) | 0.15 (0.43) | 0.15 (0.43) |
| MEM | R | -0.18 (0.35) | -0.18 (0.35) | -0.18 (0.35) | -0.18 (0.35) |
| MEM | S | 0.18 (0.35) | 0.18 (0.35) | 0.18 (0.35) | 0.18 (0.35) |
| NA | R | -0.19 (0.32) | -0.19 (0.32) | -0.19 (0.32) | -0.19 (0.32) |
| NA | S | 0.19 (0.32) | 0.19 (0.32) | 0.19 (0.32) | 0.19 (0.32) |

**Legends:** **Pearson's Coefficient** values (p-value). R= resistance, I= intermediate sensitivity, S= sensitive. AMC= amoxicillin, AK= amikacin, CAZ= ceftazidime, CPD= cefpodoxime, NOR= norfloxacin, CTR= ceftriaxone, CXM= cefixime, FEP= cefepime, CN= gentamicin, IPM= imipenem, MEM= meropenem, NA= nalidixic acid.
