# Supplementary figures and images for: First report of extended-spectrum beta lactamase (ESBL) and carbapenemase-producing MDR Klebsiella pneumoniae from Fuchka
Source: PLoS One. 2026 Jan 30;21(1):e0341583. doi: 10.1371/journal.pone.0341583 (PMC12858000; doi:10.1371/journal.pone.0341583)

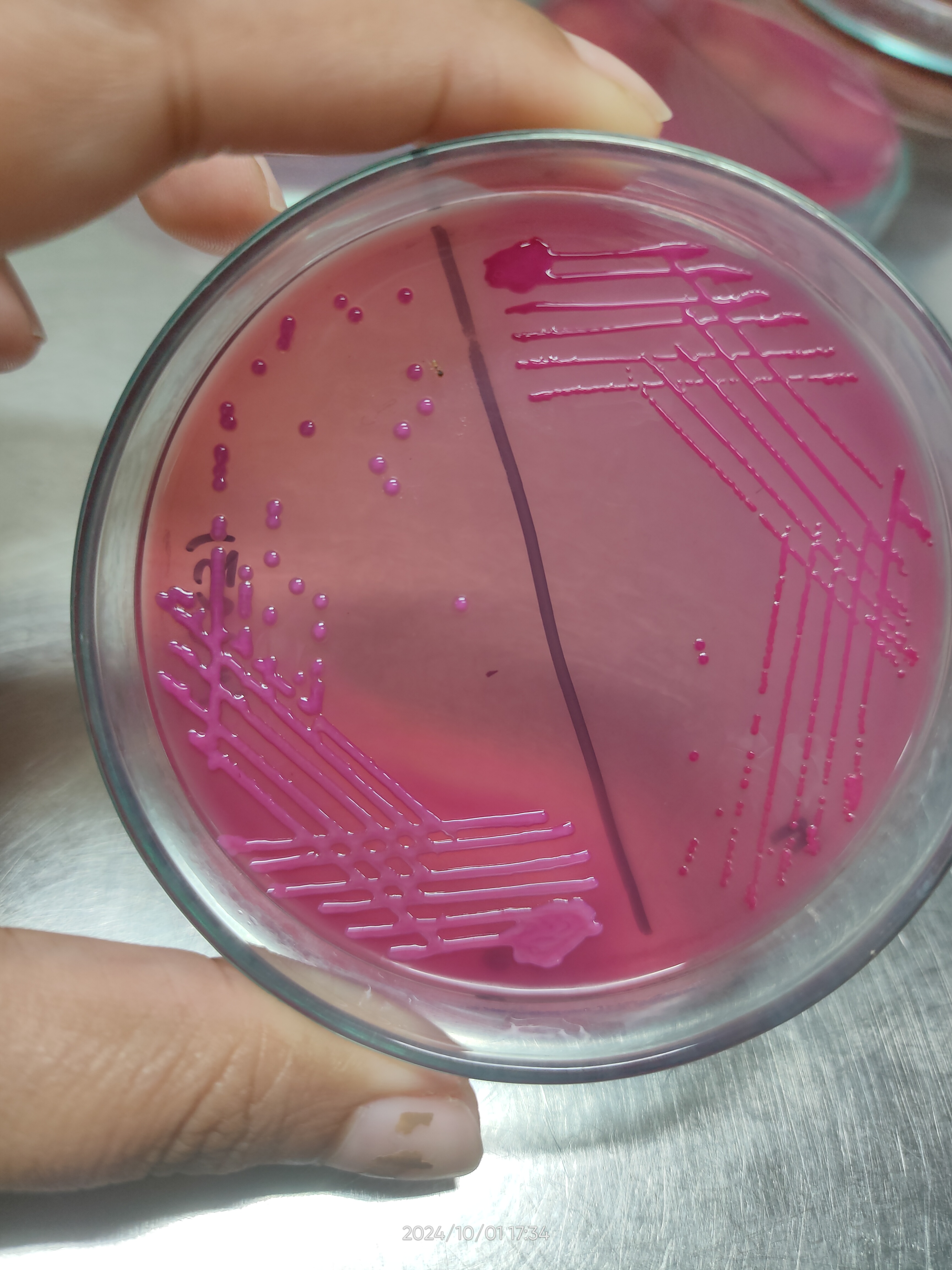

Supplement: S1 Fig — (JPG) [file pone.0341583.s003.jpg]

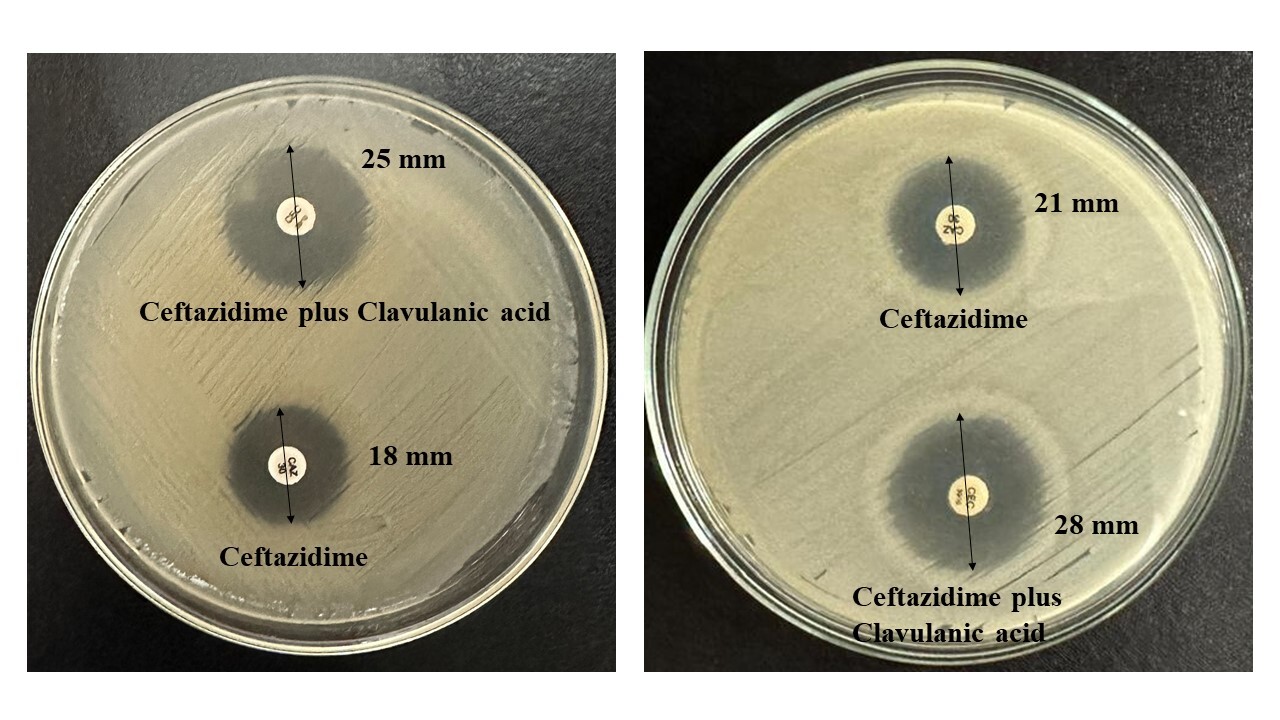

Supplement: S2 Fig — (JPG) [file pone.0341583.s004.jpg]

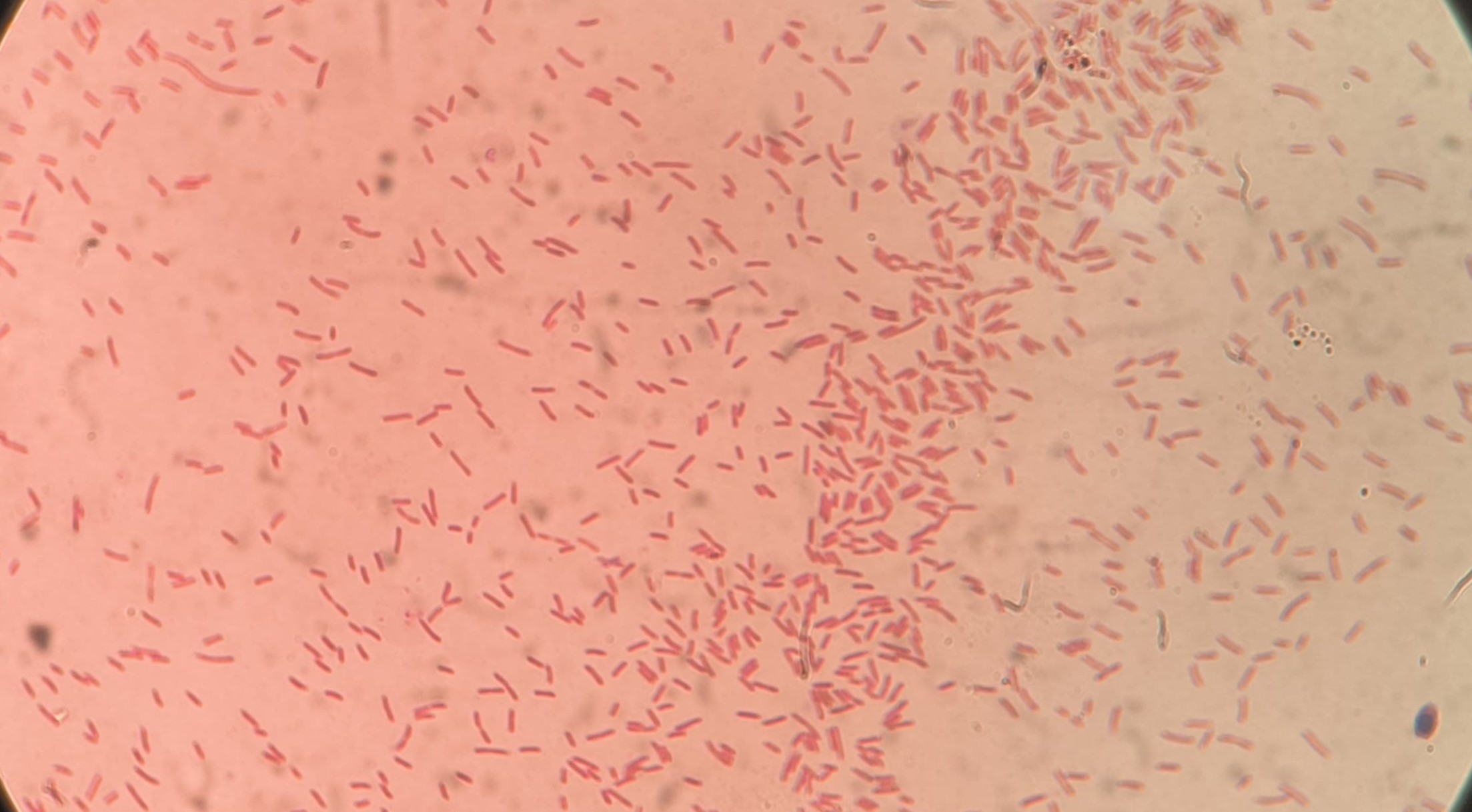

Supplement: S3 Fig — (JPG) [file pone.0341583.s005.jpg]
